# Molecular Detection of Reticuloendotheliosis Virus 5′ Long Terminal Repeat Integration in the Genome of Avipoxvirus Field Strains from Different Avian Species in Egypt

**DOI:** 10.3390/biology9090257

**Published:** 2020-08-31

**Authors:** Samah M. Mosad, Mohamed El-Tholoth, Ali A. El-Kenawy, Lina Jamil M. Abdel-Hafez, Fatma A. El-Gohary, Hanem El-Sharkawy, Mona Mohieldin Elsayed, Ayman A. Saleh, Ehab Kotb Elmahallawy

**Affiliations:** 1 Department of Virology, Faculty of Veterinary Medicine, Mansoura University, Mansoura 35516, Egypt; dr.sama786@yahoo.com (S.M.M.); tholothvirol@mans.edu.eg (M.E.-T.); elkenawya@mans.edu.eg (A.A.E.-K.); 2 Veterinary Science Division, Al Ain Men’s Campus, Higher Colleges of Technology, Al Ain 17155, UAE; 3 Department of Microbiology and Immunology, Faculty of Pharmacy, October 6 University, October 6 City 12566, Egypt; Lina.jamil@ymail.com; 4 Department of Hygiene and Zoonoses, Faculty of Veterinary Medicine, Mansoura University, Mansoura 35516, Egypt; dr.fatmagohary@gmail.com (F.A.E.-G.); dr.monamohy@yahoo.com (M.M.E.); 5 Department of Poultry and Rabbit Diseases, Faculty of Veterinary Medicine, Kafrelsheikh University, Kafrelsheikh 33511, Egypt; hanem_amin@yahoo.com; 6 Department of Animal Wealth Development, Genetics and Genetic Engineering, Faculty of Veterinary Medicine, Zagazig University, Zagazig 44519, Egypt; lateefsaleh@yahoo.com; 7 Department of Biomedical Sciences, University of Leon, 24071 León, Spain; 8 Department of Zoonoses, Faculty of Veterinary Medicine, Sohag University, Sohag 82524, Egypt

**Keywords:** *Avipoxvirus*, Egypt, *P4b* gene, *Reticuloendotheliosis* virus, 5′LTR

## Abstract

Avipoxviruses (APVs) are among the most complex viruses that infect a wide range of birds’ species. The infection by APVs is often associated with breathing and swallowing difficulties, reduced growth, decreased egg production, and high mortalities in domestic poultry. In the present study, 200 cutaneous nodular samples were collected from different avian species (chicken, pigeon, turkey, and canary) suspected to be infected with APVs from Dakahlia Governorate, Egypt. Pooled samples (*n* = 40) were prepared and inoculated in embryonated chicken eggs (ECEs). APVs were then identified by polymerase chain reaction (PCR) and sequence analysis of the APV *P4b* gene. Furthermore, the forty strains of APVs were screened for the presence of reticuloendotheliosis virus (REV)-5′LTR in their genomes. Interestingly, the phylogenic tree of the APV *P4b* gene was separated into 2 clades: clade 1, in which our fowlpox virus (FWPV), turkeypox virus (TKPV), and canarypox virus (CNPV) isolates were grouped, along with reference FWPVs and TKPVs retrieved from GenBank, whereas, in clade2, the pigeonpox virus (PGPV) isolate was grouped with PGPVs retrieved from GenBank. Likewise, REV-5′LTR was amplified from 30 strains isolated from chicken, turkey, and canary, while PGPV strains were free from REV-5′LTR integration. To the best of our knowledge, this study involved the detection and characterization of REV-5′LTR insertions in the APVs field isolates in Egypt for the first time. Given the above information, further future research seems recommended to understand the impact of the resulting REV-5′LTR insertions on the pathogenesis, virulence, and inadequate vaccine protection against APVs.

## 1. Introduction

Avipoxviruses (APVs) are a group of mosquito-borne viruses of the genus *Avipoxvirus* that belong to the family *Poxviridae,* subfamily *Chordopoxvirinae* (ChPV) [1,2]. This family of viruses *(Poxviridae*) comprises a number of zoonotic pathogens that possess public health concerns [3]. According to the Statutes of International Committee on the Taxonomy of Viruses (ICTV), APVs are divided into twelve species that got their names based on the bird species from which the virus was isolated and characterized, such as fowlpox virus, pigeonpox virus, turkeypox virus, and canarypox virus that were isolated from chickens, pigeons, turkeys, and canaries, respectively [2,4,5]. Importantly, APVs infections can result in huge economic burdens that target the poultry industry represented by a reduction in growth and egg production. Furthermore, APVs possess obvious oncogenic properties and result in a considerable increase in the mortality rate, particularly under stress conditions [4,6,7,8].

It is noteworthy to state that APV infections occur in three forms: dry form (cutaneous), wet form (diphtheritic), and systemic form, in which different body systems are involved [9,10]. In the cutaneous form, the proliferative lesions appeared as papules, which develop to nodules, then wart-like masses, which harden and form scabs on unfeathered skin areas, especially combs and wattles [4,11,12,13,14]. Regarding the diphtheritic form, opaque white necrotic fibrous proliferative slightly elevated nodules are frequently developed, then rapidly increase in size and form a yellowish diphtheritic membrane on the mucous membranes of the upper digestive and upper respiratory tracts [4,11,12,13,14]. In accordance with its epidemiological pattern, APVs are transmitted by aerosols and insect bites, mainly mosquitoes and arthropods, while the epidemiological profile of the disease includes domestic poultry, pets, and many species of wild birds [4,8,15,16]. The control of the disease is mainly based on hindering the transmission and vaccination of susceptible birds [15,16].

Importantly, APVs genome comprises a 260 to 365-Kbp double-stranded (ds)DNA genome with more than 250 putative genes. APVs replicate in the cell cytoplasm, with ChPV common regulatory elements for gene expression [17]. APVs genomes exhibit large-scale genomic rearrangements, more extensive gene families, and novel host range genes in comparison with other members of ChPV [4,16,17]. Although, there is a serologic cross-reactivity and antigenic relationship between APVs, a major difference exists in their host specificity and pathogenicity [7,18,19]. Taken into account the scarcity of the information about APV phylogeny and the difficulty in identifying the pan-genus for amplifying different genes, the APV *P4b* core protein gene, which encodes for 75.2 kDa protein, is mostly used for the molecular detection and comparative genetic analysis of APVs, as the *P4b* gene is a highly conserved gene in different APV species [20,21,22,23,24].

On the other hand, the reticuloendotheliosis virus is a neoplastic, immunosuppressive avian retrovirus. Over 50 years ago, researchers detected the reticuloendotheliosis virus sequence integration in the fowlpox virus (FWPV), pigeonpox virus (PGPV), and turkeypox virus (TKPV) genomes [25,26,27]. Full infective reticuloendotheliosis virus (REV) provirus integration in poxvirus genomes has been associated with immunosuppression, virulence enhancement, and the dissemination of REV, which occurs as a consequent exacerbated progression of the disease [25,28]. In spite of REV provirus integration in most field strains of fowlpox virus (FWPV), vaccine strains only contain long terminal repeats remnants [26]. The integration of infectious REV Provirus and LTR sequences only occur at a definite location in the FWPV genomes, which is exactly between the FWPV201 and FWPV203 genes, while the FWPV202 gene was mainly encompassed by the LTR sequences [29]. It has been reported that the FWPV vaccines are the cause of REV provirus retention in fowls, but some researchers disagree with this hypothesis, since REV integration was detected in the FWPV genome before the wide spread of the FWPV vaccine in poultry [30]. It is noteworthy to state that several previous studies across the world detected REV sequences integrated in the genomes of field and vaccine strains of FWPV, PGPV, and TKPV [16,17,31,32,33,34,35,36,37,38,39,40,41,42,43]. Still, no REV full or partial LTRs have never seen integrated in canarypox viruses (CNPVs) [16,17,31,32,33,34,35,36,37].

Taken into account, the acquired retroviral genes expression can alter the host’s biological properties and results in a new modified virus emergency and the currently used vaccines, which in turn leads to inadequate protection and sometimes causes recurring infections [38,39,44]. It should be stressed that FWPV strains carrying REV sequences are more pathogenic to poultry [45]. Although several studies have been conducted on APVs in Egypt [44,46,47,48,49], little is yet known about the integration of REV in the genome of APV field isolates in our country. To our knowledge, only one study has explored REV as a contaminant of fowlpox vaccines in Egypt [37]. Taken together, the present study targets the molecular identification and differentiation between APVs from different hosts (chickens, turkeys, pigeons, and canaries), depending on the *P4b* gene. Our study also involved the molecular detection of REV-5′LTR insertions in the genome of field strains of APVs infecting different avian species in Egypt.

## 2. Material and Methods

### 2.1. Ethical Consideration

Ethical approval was obtained from a guidance of Research, Publication, and Ethics of the Faculty of Veterinary Medicine, Mansoura University, Egypt, which complies with all relevant Egyptian legislations.

### 2.2. Study Area, Samples Collection, and Preparation

Clinical samples of proliferative cutaneous nodular lesions (*n* = 200) were collected from different avian species, including chickens, pigeons, turkeys, and canaries from Dakahlia Governorate, Egypt during the period from January to December 2017. These samples were collected from birds suspected to be infected with APVs (Table 1).

Taken into consideration, the sampling and samples size were performed according to a protocol carried out by Cannon and Roe (1982) [50]. Each five nodular samples from the same bird were pooled together. Forty pooled samples were created (10 chicken, 10 pigeon, 10 turkey, and 10 canary samples). The control negative sample was included and pooled from the skin of 5 normal chickens. Collected cutaneous samples were then homogenized with phosphate-buffered saline (PBS) containing antibiotics (penicillin 50 IU and streptomycin 50 µg per mL) in sterile mortar, and 10% (W/V) suspensions were prepared. Then, suspensions were centrifuged for 15 min at 4000 rpm in cooling centrifuge, and supernatants were collected and confirmed to be free from REV coinfection by real-time PCR, as previously described [51], then stored at −20 °C until use for APV isolation [22,52].

### 2.3. Standard FWPV

The standard FWPV was the DECP25 strain of the FWPV vaccine (Diftosec, MERIAL, Athens, GA, USA, Batch no: L450985). Each vial contained 10^6^ TCID_50_ FWPV, which reconstituted in 5-mL PBS and was used as a control positive in PCR.

### 2.4. Embryonated Chicken Eggs (ECEs)

Nine-day-old SPF ECEs were purchased from Egyptian S.P.F. Eggs Production Farm (Nile SPF), Fayoum, Egypt. At day 10, twenty eggs were randomly selected and confirmed to be free from vertically transmitted REV contamination by real-time PCR, as previously described elsewhere [51].

### 2.5. Virus Isolation

The supernatant fluid from each sample was inoculated into 11-day-old REV-free SPF ECE (0.2 mL/ECE) via the chorioallantoic membrane (CAM) route. Inoculated eggs were incubated for 6 days at 35 °C; then, the CAMs were collected and examined for specific pock lesions, followed by the collection of CAMs containing pock lesions. A single separate pock was then picked from the CAM from each sample, homogenized with PBS, centrifuged, and the supernatant fluids were stored at −20 °C until their use in PCR [22].

### 2.6. Molecular Detection of the Isolated Virus

#### 2.6.1. DNA Extraction

QIAamp^®^ MinElut^®^ Virus Spin Kit (QIAGEN, GmbH, Hilden Germany) was used for the extraction of DNA from 40 tested isolates (homogenates from separate pock lesions) using control negative (uninfected chicken skins) and control positive samples (FWPV DECP25 strain), according to the kit’s guidelines. Briefly, 200 µL of supernatant fluid from each isolate was added to 20-µL proteinase K enzyme; then, 200 µL of lysis buffer was added and mixed by pulse vortex for 10 s. The mixture was incubated at 56 °C for 15 min; then, 200 µL of pure ethanol was added, and the mixture was incubated at room temperature for 5 min. The mixture was then loaded in the spin column; centrifuged for 1 min at 8000 rpm; and washed 3 times with washing buffer 1, washing buffer 2, and pure ethanol (500 µL each). The membranes were then dried well by centrifugation at 12,000 rpm for 2 min, then incubated with an opened lid at 56 °C for 5 min. The viral DNA was then eluted with 50-µL elution buffer in DNase/RNase-free Eppendorf.

#### 2.6.2. Polymerase Chain Reaction

Two PCR protocols were applied: the first protocol was used for the detection of APVs by amplification of the *Avipoxvirus P4b* core protein gene (fpv167 locus). The other PCR protocol was used for the detection of REV integration through the amplification of APV genome regions flanking the REV-5′LTR integration site. Taken into consideration, REV integration was detected with REV-FWPV heterologous primer pair with forward primer that annealed to the FWPV 201 gene at nucleotide no. 231,521 to 321,540 and reverse primer that annealed to REV 5′LTR at nucleotide no. 231,874 to 231,891 of the reference APV MH719203/TKPV SD15-670.1 isolate [32]. Both PCR protocols were done with the extracted DNA from separate pock lesions homogenates of 40 tested isolates (10 FWPV, 10 PGPV, 10 TKPV, and 10 CNPV). The PCR protocols were performed using two sets of primers according to protocols previously described elsewhere [53,54]. These primers were obtained from Metabion International AG, Planegg, Germany and are shown in Table 2. The reaction mixture included 25-μL Dream Taq Green PCR Master Mix (2X) (Thermo Scientific, Waltham, MA, USA), 4-μL DNA, 1-μL (10-pmol) forward and reverse primers for each gene, and nuclease-free water up to 50 μL. A control positive (FWPV DECP25 strain) and a control negative (noninfected chicken) reaction were included in each cycle.

The PCR protocol was performed in a thermal cycler (Biometra T-Gradient, Göttingen, Germany) as follows: a single cycle of initial denaturation at 94 °C for 2 min, followed by 25 cycles of denaturation at 94 °C/1 min, annealing at 61 °C (in the case of the *P4b* gene) or 52 °C (for 5′LTR) for 1 min, and the reaction was completed by a final extension at 72 °C for 1 min, with a final incubation step at 72 °C for 10 min. Then, 1.5% agarose gel in 0.5% ((Sigma-Aldrich, Cairo, Egypt) Tris-Borate EDTA buffer (Sigma-Aldrich, Cairo, Egypt) was used for separation of the PCR products with a 100-bp DNA ladder (Jena Bioscience, Jena, Germany). DNA bands were visualized with a UV transilluminator.

### 2.7. Sequencing of APV P4b Gene and REV 5′LTR

Four PCR products represent the positive samples from each APV type (a total of 16 samples) and were selected for nucleotide sequencing. These samples were selected based on the intensity of the band of the PCR product in the gel and from flocks with high mortality rates (20–50%). Nucleotide sequencing was on amplified DNA from both field samples and viruses’ isolates. The amplified DNA bands of the APV *P4b* gene (578 bp) and REV 5′LTR (370 bp) were then excised and purified from the gel with QIAquick PCR gel purification kit (Qiagen Inc., Valencia, CA, USA) according to the kit’s guidance. The purified PCR products were then transported to Macrogen Clinical Laboratory (Korea) for DNA sequencing (bidirectional) using the same primer sets used in the conventional PCR. The obtained nucleotide sequences (Table 3) were deposited in the GenBank (http://www.ncbi.nlm.nih.gov/Genbank).

### 2.8. Sequencing Data Analysis

Analysis of the obtained sequence data was performed via ClustalW2 version 2.1 (https://www.ebi.ac.uk/Tools/msa/clustalw2/). The alignment output files were used for conducting the phylogenetic Neighbor joining (NJ) and bootstrap NJ analyses, with 1000 repeat bootstrap tests in MEGA X software version 2 (http://www.megasoftware.net/).

## 3. Results

### 3.1. Clinical Signs and Postmortem Changes

**Figure 1:**
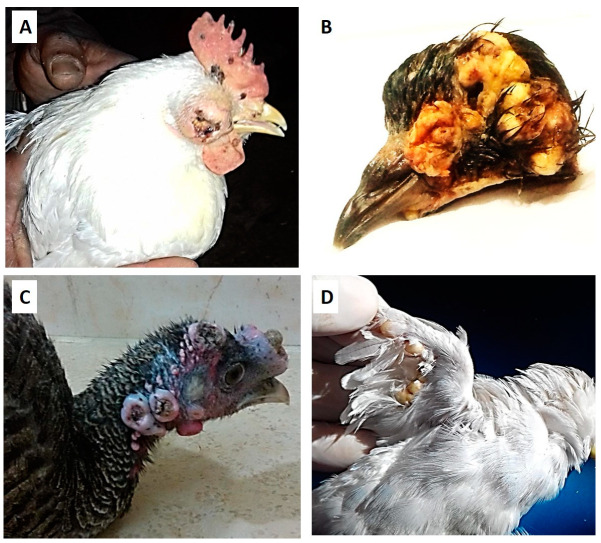
Avipoxvirus (APV) lesions in a chicken, pigeon, turkey, and canary. (**A**) Fowlpox virus (FWPV) lesions exist on the comb and wattles and occupy the eye of affected white leghorn chicken. (**B**) Pigeonpox virus (PGPV) proliferative lesions on the head and wings of affected Egyptian swift pigeon. (**C**) Multiple turkeypox virus (TKPV) lesions on the head of a diseased bronze turkey, and the snood was totally involved with a pox lesion. (**D**) Canarypox virus (CNPV) lesions on the wing of diseased white canary.

Regarding the clinical signs in chickens, pox lesions were observed on the legs, wings, comb, wattles, and head, especially around the eye, with some birds showing complete involvement of the eye, causing permanent loss of the eyes (Figure 1A).

On the other hand, proliferative lesions were observed on the wings and head in pigeons (Figure 1B), while, in turkeys, most lesions were observed on the head, and the snood was totally involved, and pox lesions were also observed under the wings and in the vent area (Figure 1C). The affected canaries had some pox lesions on the head and legs, and most lesions were observed on the wings (Figure 1D).

### 3.2. Virus Isolation

**Figure 2:**
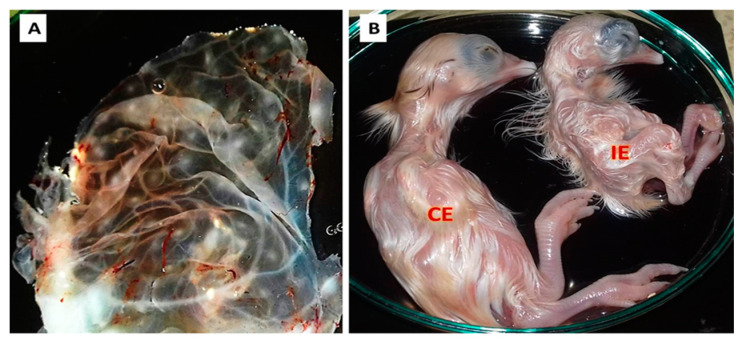
Result of APV isolation on the chorioallantoic membranes (CAMs) of 11-day-old embryonated chicken eggs (ECEs). (**A**) Pock lesions distributed on the CAMs of inoculated ECEs. (**B**) Control embryo (CE), which was inoculated with the control negative sample, and the APV inoculated embryo (IE) showing dwarfism and abnormal feathering.

All inoculated samples (*n* = 40) and the control positive sample showed clear pock lesions on the CAM of 11-day-old SPF ECEs, which is shown in Figure 2A, with dwarfed, abnormal feathered embryos (Figure 2B) and mortality in some inoculated embryos five days post-inoculation. On the other hand, the control negative sample showed no changes in the inoculated eggs.

### 3.3. Molecular Identification Using Conventional PCR

In the present work, the amplified fragment size for the targeted genes (*P4b* gene) of the tested isolates (10 FWPV, 10 PGPV, 10 TKPV, and 10 CNPV) was around 578 bp, which is nearly similar to that of the control positive. On the other hand, out of 40 REV-5′LTR tested isolates, 30 isolates (10 FWPV, 10 TKPV, and 10 CNPV isolates) were confirmed to have REV-5′LTR integration in their genome, with an amplicon of 370 bp, which is similar to that of the positive control. Moreover, the remaining 10 PGPV tested isolates that expressed APV-*P4b* gene amplification did not show any amplified bands for REV-5′LTR. This result confirms that the PGPV isolates were free from REV integration, which is similar to that of the negative control samples.

### 3.4. Sequencing and Phylogenetic Analysis of the APV P4b Gene and REV- 5′LTR

**Figure 3:**
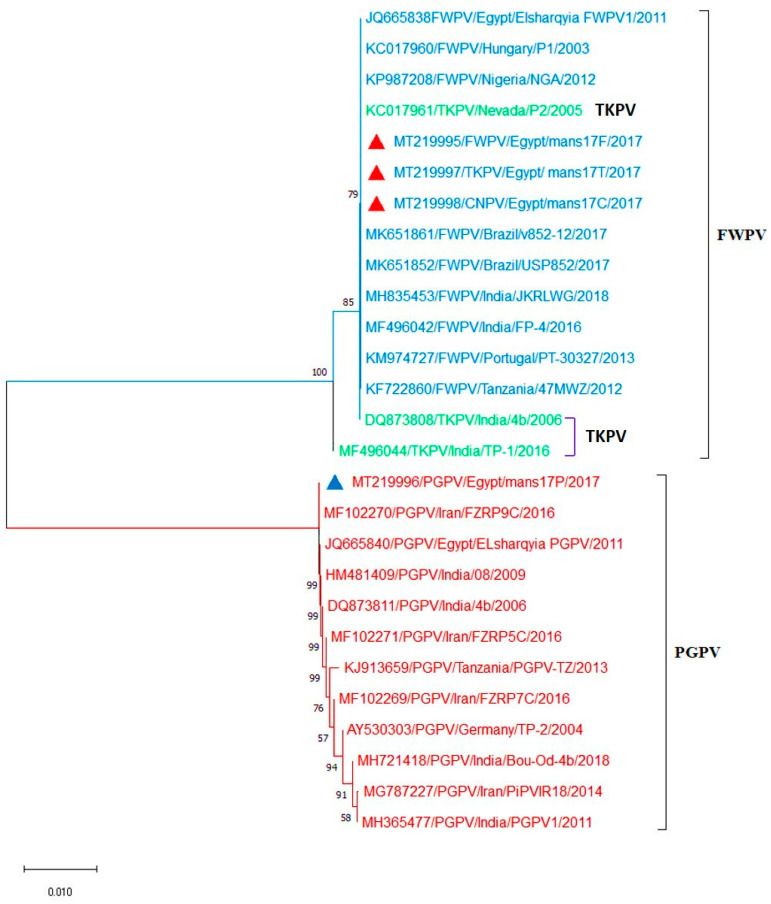
Phylogenetic Neighbor-Joining tree of the APV *P4b* gene sequences with 1000 repeats bootstrap. The tree separated into 2 clades: clade 1, in which the isolates mans17F (FWPV), mans17T (TKPV), and mans17C (CNPV) identified in this study (red triangles) are aligned with FWPVs and TKPVs obtained from GenBank. Clade2, in which the mans17P (PGPV) isolate identified in this study (blue triangle) is aligned with PGPVs retrieved from GenBank.

**Figure 4:**
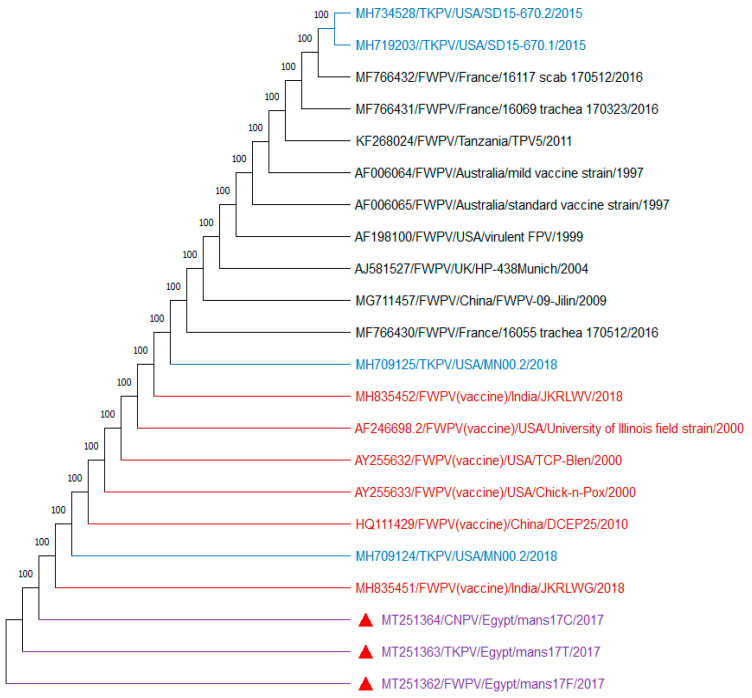
Phylogenetic Neighbor-Joining tree of REV-5′LTR sequences with 1000 repeats bootstrap. The mans17F (FWPV), mans17T (TKPV), and mans17C (CNPV) identified in this study (red triangles) are identical to other REV-5′LTR integrated in FWPV (field-isolated and vaccine strains) and TKPV isolates obtained from GenBank.

Interestingly, the amplified DNA bands of the APV *P4b* gene (578 bp) and REV 5′LTR (370 bp) were subjected to DNA sequencing. The obtained sequences were then transported to GenBank and analyzed with respect to the reference *P4b* gene and REV-5′LTR sequences from GenBank (Figure 3 and Figure 4).

Sequencing of the amplified DNA showed 100% identity between samples selected from each APV type. Importantly, no difference was recorded among the nucleotides sequences of amplified DNA obtained from the field samples and virus isolates. Interestingly, the APV *P4b* gene phylogenic tree was separated into 2 clades: clade 1, in which FWPV (mans17F), TKPV (mans17T), and CNPV (mans17C) isolates identified in this study were aligned with FWPVs and TKPVs obtained from GenBank. The isolates mans17F, mans17T, and mans17C were identical to each other, and they showed 100% identity to other FWPVs and TKPVs obtained from GenBank. Whereas, in clade 2, PGPV, the (mans17P) isolate was aligned with other PGPVs retrieved from GenBank. The isolate mans17P showed 100% identity to other PGPVs obtained from GenBank (Figure 3). The PGPV isolate (mans17P) identified in the present study recorded 91.7% identity with FWPV (mans17F), TKPV (mans17T), and CNPV (mans17C), with 44 nucleotide substitutions (Supplementary Figure S1). On the other hand, as shown in Figure 4, REV-5′LTR sequences identified in this study from mans17F, mans17T, and mans17C isolates showed 100% identity to other REV-5′LTR integrated in FWPV (field-isolated and vaccine strains) and TKPV isolates obtained from GenBank (Supplementary Figure S2). Collectively, the studied FWPV, TKPV, and CNPV were identical to each other and had REV- 5′LTR integration, while the PGPV was distinct and confirmed to be free from REV- 5′LTR integration and aligned in a separate clade from FWPV, TKPV, and CNPV.

**Figure 5:**
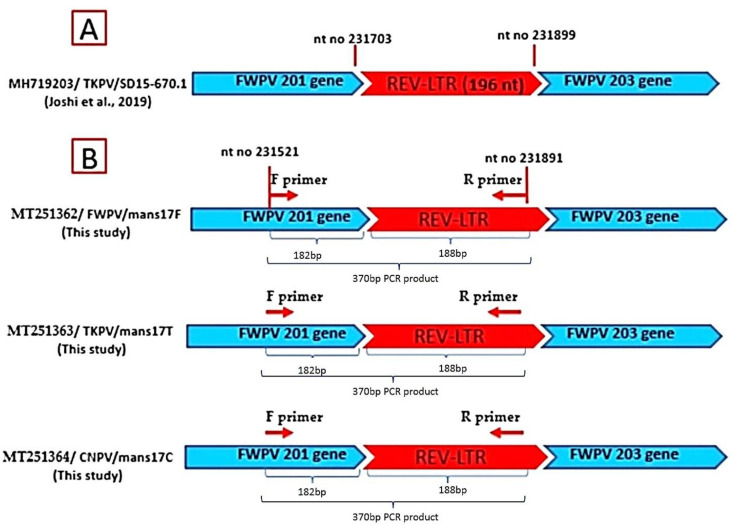
Schematic representation of REV-5′LTR insertion in the APV strains identified in this study in comparison with the TKPV-SD15-670.1 reference strain. (**A**) TKPV-SD15-670.1 reference strain containing 196 nt of REV-5′LTR between the FWPV 201 and 203 genes (from nucleotide no. 231703 and 231899). (**B**) Schematic representation of REV-5′LTR insertion in mans17F, mans17T, and mans17C isolates. The REV-5′LTR product (370 bp) falls between nucleotide no. 231521 and 231891 when aligned with SD15-670.1. This product includes 182 bp of the FWPV 201 gene, and the other 188 bp represents the inserted REV-5′LTR.

Importantly, the obtained REV-5′LTR sequences (370 bp) from mans17F, mans17T, and mans17C isolates were aligned with the full-genome sequence of the MH719203/TKPV SD15-670.1 isolate as a reference APV [32], using the NCBI BLAST tool that was used to detect the insertion site of REV in the APV genomes. The results revealed that the amplified 370-bp product falls between nucleotide no. 231,521 of the FWPV 201 gene and nucleotide no. 231,891 of REV-5′LTR when aligned with the SD15-670.1 strain. This product includes 182 bp of the FWPV 201 gene, and the other 188 bp represents the inserted REV-5′LTR, which confirms REV insertion mans17F, mans17T, and mans17C isolate genomes, as shown in Figure 5.

## 4. Discussion

The present data provides novel interesting findings in relation to the occurrence of APVs on 40 cutaneous pooled samples from Dakahlia Province, Egypt. The work also included screening same samples for the presence of REV-5′LTR in their genomes, which has been confirmed through molecular identification and a sequence analysis. To the best of our knowledge, this study is the first report involved in the detection and characterization of REV-5′LTR insertions in the APV field strains in Egypt. As depicted in our results, the examined birds showed some clinical signs cohered with APV infections, and these findings are in harmony with those reported elsewhere [4,9,11,13,14,48,55,56,57,58]. Importantly, APVs were successfully isolated on the CAM of 11-day-old SPF REV-free ECEs from 40 tested samples, and these ECEs had characteristic pock lesions with dwarfed embryos and mortality in some inoculated embryos five days post-inoculation. These results are consistent with that reported in a previous study [22].

The molecular detection using PCR is widely accepted as a sensitive, specific, and rapid molecular tool for the detection of APV genomes [46,59]. In this regard, several studies have documented the isolation of APVs from poultry in Egypt [44,48,49]. Our present molecular findings revealed that the APV *P4b* gene was successfully amplified from all 40 tested isolates, which is in agreement with that obtained in previous studies [21,22]. Furthermore, the analysis of the sequence of the APV *P4b* gene revealed that the isolates mans17F, mans17T, and mans17C were identical to each other and to other FWPVs and TKPVs obtained from GenBank, with 100% identity. In the same line, the sequence analysis of our APV *P4b* gene showed 100% identical DNA sequences to that reported in a previous study [59], while showing 71–100% similarity in nucleotide sequences between different species of APVs [22,24]. However, FWPV and TKPV seem to be identical, as they produced identical restriction patterns [25]; some researchers suggested that the TKPV is a different strain based on the difference in genome sizes (288.54 Kbp for FWPV versus 188.53 Kbp for TKPV) and the susceptibility of ducks to TKPV but not FWPV [60,61,62,63]. Additionally, the difference in physical and chemical properties, cross-protection, and phylogenetic analysis might support the theory that suggests that the TKPV is different from FWPV [17,39,64,65]. In accordance with PGPV, it was considered to be different from FWPV, as they showed host specificity and different profiles with the nucleotide sequence analysis of the *P4b* gene [24,25,66]. Interestingly, the mans17P isolate identified in the present study showed 100% identity to other PGPVs obtained from GenBank, while the PGPV identified in this study was different from FWPV (mans17F), TKPV (mans17T), and CNPV (mans17C), with 91.7% identity and 44 nucleotide substitutions [4,22,44].

In the present study, REV-5′LTR was successfully amplified in 30 isolates (10 FWPV, 10 TKPV, and 10 CNPV), confirming the integration of REV-5′LTR into FWPV, TKPV, and CNPV. Similar results were reported by Masola et al. (2014) [67], who confirmed the integration of REV in 53 out of 55 tested FWPV isolates. In other studies [25], REV-5′LTR was detected in FWPV and TKPV genomes. Similar results were reported in relation to the integration of REV sequences into the FWPV and TKPV genomes [54,68,69]. On the other hand, Davidson et al., 2008 [35], found that seven APV isolates (two chickens, one turkey, and four wild birds) were free from any REV integration. In addition, several previous studies suggested that CNPV field strains and CNPV vaccine strains were free from REV-5′LTR [17,30,40]. On the other hand, our present results showed that PGPV isolates were free from the integration of REV-5′LTR in their genomes, which is in stark contrast with previous studies that reported that REV-5′LTR integration was detected in PGPV [25]. Kim and Tripathy, 2001 [40], revealed that PGPV was free from REV-5′LTR integration. In accordance with the sequence analysis, the identified sequence of REV-5′LTR in the current study of mans17F, mans17T, and mans17C isolates showed 100% identity with the other REV-5′LTR integrated in FWPV field-isolated and vaccine strains and TKPV isolates [27,70]. Collectively, the present findings provide interesting information in relation to the potential impacts of infection with these viruses in poultry, as they may result in tumorigenic and immunosuppressive diseases [71,72]. Despite the absence of the difference among the nucleotides sequences of the amplified DNA obtained from the field samples (lesions) and virus isolates in our present results, it is recommended to perform a sequence analysis directly from the DNA purified from the lesion to avoid the possibility of changes and the exclusion of REV genomic fragments from the APV genomes during additional reamplification in ECEs.

## 5. Conclusions

Given the above information, our data reveal that APVs are circulating in Egypt and infecting different avian species. The present study also provides interesting information about the genetic relatedness of these viruses. Furthermore, the present molecular data indicate that FWPV, TKPV, and CNPV were identical to each other and different from PGPV. Importantly, REV-5′LTR was detected in FWPV, TKPV, and CNPV, while PGPV was free from REV -5′LTR integration. Taken into account the shortage of data about the studied topic and the possibility of coinfection of both APVs and REV, further future studies are crucial to investigate the effect of REV -5′LTR insertions in APV strains on the pathogenesis, virulence, and inadequate vaccine protection against APVs in Egypt. There is also a need to investigate the epidemiological profile of these viruses in the Egyptian environment and the effect of REV-5′LTR insertion on the acquisition of evolutionary features over time in certain regions of APV genomes.

## Figures and Tables

**Table 1:** Details of collected samples, including host, breed, number of collected samples, vaccination, morbidity, mortality rates, and lesion distributions.

Host	Chicken	Pigeon	Turkey	Canary
**Breed(s)**	White leghorn	Egyptian Swift	Bronze Turkey	white canary and yellow canary
No. of collected samples	10	10	10	10 (6 white canaries, 4 yellow canaries)
Vaccination	4/10 vaccinated 6/10 not vaccinated	Not vaccinated	Not vaccinated	Not vaccinated
Morbidity rate	70–100%	80–100%	80–100%	75–100%
Mortality rate	0–25%	15–30%	0–20%	20–50%
Pox lesions distribution	Legs, wings, comb, wattles, and head, especially around the eye	on the head and wings	on the head, under the wings, and vent areas	All over the body, especially on the head and wings

**Table 2:** Details of the two sets of primers that were used for amplification of the APV *P4b* gene and REV 5′LTR.

Primer	Sequence (5′– 3′) Direction	Target	Product Size (bp)	Reference
APV *P4b* forward	CAGCAGGTGCTAAACAACAA	APV-*P4b* gene	578	[53]
APV *P4b* reverse	CGGTAGCTTAACGCCGAATA			
REV 5′LTR forward	ACCTATGCCTCTTATTCCAC	REV-5′LTR	370	[54]
REV 5′LTR reverse	CTGATGCTTGCCTTCAAC			

**Table 3:** Details of the APV *P4b* gene and REV 5′LTR DNA sequences, including virus species, host, isolate, and accession numbers of the *P4b* gene and REV-5′LTR nucleotide sequences. FWPV: fowlpox virus, PGPV: pigeonpox virus, TKPV: turkeypox virus, and CNPV: canarypox virus.

Virus Species	Host	Isolate	P4b Gene Accession Number	REV-5′LTR Accession Number
FWPV	Chicken	mans17F	MT219995	MT251362
PGPV	Pigeon	mans17P	MT219996	-------------
TKPV	Turkey	mans17T	MT219997	MT251363
CNPV	Canary	mans17C	MT219998	MT251364

## Supplementary Materials

The following are available online at https://www.mdpi.com/2079-7737/9/9/257/s1: Figure S1: The BioEdit multiple alignments of amplified *P4b* gene nucleotide sequences of our APVs isolates compared to other reference APVs sequences retrieved from GenBank. PGPV strains have 44 nucleotide substitutions in comparison with FWPV and TKPV strains. Supplementary Figure S2: The BioEdit multiple alignments of amplified REV-5′LTR nucleotide sequences detected in our APVs isolates in comparison to other reference REV-5′LTR sequences available in GenBank. All aligned sequences are identical to each other without any nucleotide substitution.

## References

[1] Fauquet C.M. (2005). Virus Taxonomy: VIIIth Report of the International Committee on Taxonomy of Viruses.

[2] CTV (2012). ICTVdB Index of Viruses. International Committee on Taxonomy of Viruses. http://ictvonline.org/.

[3] Essbauer S., Pfeffer M., Meyer H. (2010). Zoonotic poxviruses. Veter. Microbiol..

[4] Weli S.C., Tryland M. (2011). Avipoxviruses: Infection biology and their use as vaccine vectors. Virol. J..

[5] Virus Taxonomy 2019 Release. International Committee on Taxonomy of Viruses. talk.ictvonline.org.

[6] Pledger A. (2005). Avian pox virus infection in a mourning dove. Can. Veter J. La Rev. Veter. Can..

[7] Bolte A.L., Meurer J., Kaleta E.F. (1999). Avian host spectrum of avipoxviruses. Avian Pathol..

[8] Ritchie B.W. (1995). Avian Viruses: Function and Control.

[9] Kulich P., Roubalova E., Dubská L.Z., Sychra O., Smíd B., Literak I. (2008). Avipoxvirus in blackcaps (*Sylvia atricapilla*). Avian Pathol..

[10] Atkinson C.T. (2010). Efficacy of Commercial Canarypox Vaccine for Protecting Hawaii Amakihi from Field Isolates of Avipoxvirus.

[11] Fenner F., Bachmann P.A., Gibbs E.P.J., Murphy F.A., Studdert M.J., White D.O. (1993). Veterinary Virology.

[12] OIE (2008). Manual of Diagnostic Tests and Vaccines for Terrestrial Animals.

[13] Thiel T., Whiteman N.K., Tirapé A., Baquero M.I., Cedeño V., Walsh T., Uzcátegui G.J., Parker P.G. (2005). Characterization of canarypox-like viruses infecting endemic birds in the galápagos islands. J. Wildl. Dis..

[14] Atkinson C.T., Wiegand K.C., Triglia D., Jarvi S.I. (2012). Reversion to virulence and efficacy of an attenuated canarypox vaccine in Hawaii amakihi (hemignathus virens). J. Zoo Wildl. Med..

[15] Docherty D.E., Long R.I.R., Flickinger E.L., Locke L.N. (1991). Isolation of Poxvirus from Debilitating Cutaneous Lesions on Four Immature Grackles (Quiscalus sp.). Avian Dis..

[16] Boyle D.B. (2007). Genus avipoxvirus. Poxviruses.

[17] Tulman E.R., Afonso C.L., Lu Z., Zsak L., Kutish G.F., Rock D.L. (2004). The genome of canarypox virus. J. Virol..

[18] Kim T.J., Schnitzlein W.M., McAloose D., Pessier A.P., Tripathy D.N. (2003). Characterization of an avianpox virus isolated from an Andean condor (Vultur gryphus). Veter. Microbiol..

[19] Pattison M., McMullin B., Alexander D. (2008). Poultry Diseases.

[20] Pérez-Tris J., Williams R.A., Abel-Fernández E., Barreiro J., Conesa J.J., Figuerola J., Martinez-Martínez M., Ramirez A., Benitez L. (2011). A Multiplex PCR for detection of poxvirus and papillomavirus in cutaneous warts from live birds and museum skins. Avian Dis. Dig..

[21] Manarolla G., Pisoni G., Sironi G., Rampin T. (2010). Molecular biological characterization of avian poxvirus strains isolated from different avian species. Veter. Microbiol..

[22] Fasaei N. (2014). Molecular detection and phylogenetic analysis of Avipoxvirus strains isolated from different bird species. Iran. J. Vet. Res..

[23] Weli S.C., Traavik T., Tryland M., Coucheron D.H. (2004). Analysis and comparison of the 4b core protein gene of avipoxviruses from wild birds: Evidence for interspecies spatial phylogenetic variation. Arch. Virol..

[24] Lüschow D., Hoffmann T., Hafez H.M. (2004). Differentiation of avian poxvirus strains on the basis of nucleotide sequences of 4b gene fragment. Avian Dis..

[25] Puro K.U., Ahuja A., Joishy T., Sen A., Ghatak S., Shakuntala I., Das S., Sunjukta R. (2015). Molecular detection of reticuloendotheliosis virus (REV) integration in avian poxvirus in North Eastern India. Proc. Natl. Acad. Sci. India Sect. B: Biol. Sci..

[26] Singh P., Schnitzlein W.M., Tripathy D.N. (2003). Reticuloendotheliosis Virus sequences within the genomes of field strains of fowlpox virus display variability. J. Virol..

[27] Singh P., Kim T., Tripathy D.N. (2000). Re-emerging fowlpox: Evaluation of isolates from vaccinated flocks. Avian Pathol..

[28] Walker M.H., Rup B.J., Rubin A.S., Bose H.R. (1983). Specificity in the immunosuppression induced by avian reticuloendotheliosis virus. Infect. Immun..

[29] Laidlaw S.M., Skinner M.A. (2004). Comparison of the genome sequence of FP9, an attenuated, tissue culture-adapted European strain of Fowlpox virus, with those of virulent American and European viruses. J. Gen. Virol..

[30] Moore K.M., Davis J.R., Sato T., Yasuda A. (2001). Reticuloendotheliosis virus (REV) long terminal repeats incorporated in the genomes of commercial fowl poxvirus vaccines and pigeon poxviruses without indication of the presence of infectious REV. Avian Dis..

[31] Singh P., Schnitzlein W.M., Tripathy D.N. (2005). Construction and characterization of a fowlpox virus field isolate whose genome lacks reticuloendotheliosis provirus nucleotide sequences. Avian Dis..

[32] Joshi L.R., Bauermann F.V., Hain K.S., Kutish G.F., Armién A.G., Lehman C.P., Neiger R., Afonso C.L., Tripathy D.N., Diel D.G. (2019). Detection of Fowlpox virus carrying distinct genome segments of Reticuloendotheliosis virus. Virus Res..

[33] Meroz M. (1992). Reticuloendotheliosis and ’pullet disease’ in Israel. Veter. Rec..

[34] Okoye J.O.A., Ezema W., Agoha J.N. (1993). Naturally occurring clinical reticuloendotheliosis in Turkeys and chickens. Avian Pathol..

[35] Davidson I., Shkoda I., Perk S. (2008). Integration of the reticuloendotheliosis virus envelope gene into the poultry fowlpox virus genome is not universal. J. Gen. Virol..

[36] AlFaki S.H., Hussien M.O., Osman N.A., Enan K.A., El Hussein A.R.M. (2020). First report on molecular characterization and phylogenetic analysis of Reticuloendotheliosis virus in Sudan. Trop. Anim. Health Prod..

[37] Awad A.M., El-Hamid H.S.A., Abourawash A., Ibrahim H.H. (2010). Detection of reticuloendotheliosis virus as a contaminant of fowl pox vaccines. Poult. Sci..

[38] Hertig C., Coupar B.E., Gould A.R., Boyle D.B. (1997). Field and vaccine strains of fowlpox virus carry integrated sequences from the avian retrovirus, reticuloendotheliosis virus. Virology.

[39] García M., Narang N., Reed W.M., Fadly A. (2003). Molecular characterization of reticuloendotheliosis virus insertions in the genome of field and vaccine strains of fowl poxvirus. Avian Dis..

[40] Kim T., Tripathy D.N. (2001). Reticuloendotheliosis virus integration in the fowl poxvirus genome: Not a recent event. Avian Dis..

[41] Tadese T., Reed W.M. (2003). Detection of specific reticuloendotheliosis virus sequence and protein from REV-integrated fowlpox virus strains. J. Virol. Methods.

[42] Fadly A.M., Witter R.L., Smith E.J., Silva R.F., Reed W.M., Hoerr F.J., Putnam M.R. (1996). An outbreak of lymphomas in commercial broiler breeder chickens vaccinated with a fowlpox vaccine contaminated with reticuloendotheliosis virus. Avian Pathol..

[43] Rajasekaran R. (2019). Molecular detection of integrated reticuloendothelial virus genes in fowlpox virus field isolates and live vaccines of poultry. Indian J. Anim. Sci..

[44] Lebdah M.A., Hassanin O.A., Ali A.M. (2019). Avipoxvirus in Egypt and African continent: A review. Zagazig Veter. J..

[45] Tadese T., Fitzgerald S., Reed W.M. (2008). Detection and differentiation of re-emerging fowlpox virus (FWPV) strains carrying integrated reticuloendotheliosis virus (FWPV-REV) by real-time PCR. Veter. Microbiol..

[46] Mosad S. (2019). Conventional and molecular detection of avipoxviruses from chickens, pigeons and turkeys. Mansoura Veter. Med. J..

[47] Ali A.M., Fathy A., Eid A. (2014). Detection of Avian Poxvirus in an Egyptian Goose. Glob. Anim. Sci. J.-GASJ.

[48] Abdallah F., Hassanin O. (2012). Detection and molecular characterization of avipoxviruses isolated from different avian species in Egypt. Virus Genes.

[49] El-Mahdy S.S., Awaad M.H.H., Soliman Y. (2014). Molecular identification of local field isolated fowl pox virus strain from Giza governorate of Egypt. Veter. World.

[50] Cannon R.M., Cannon R.M., Roe R.T. (1982). Livestock Disease Surveys: A Field Manual for Veterinarians.

[51] Li K., Gao H., Gao L., Qi X., Qin L., Gao Y., Xu Y., Wang X. (2012). Development of TaqMan real-time PCR assay for detection and quantitation of reticuloendotheliosis virus. J. Virol. Methods.

[52] Diallo I.S., Taylor J., Gibson J., Hoad J., De Jong A., Hewitson G., Corney B.G., Rodwell B.J. (2010). Diagnosis of a naturally occurring dual infection of layer chickens with fowlpox virus and gallid herpesvirus 1 (infectious laryngotracheitis virus). Avian Pathol..

[53] Lee L.H., Lee K.H. (1997). Application of the polymerase chain reaction for the diagnosis of fowl poxvirus infection. J. Virol. Methods.

[54] Biswas S.K., Jana C., Chand K., Rehman W., Mondal B. (2011). Detection of fowl poxvirus integrated with reticuloendotheliosis virus sequences from an outbreak in backyard chickens in India. Veter. Ital..

[55] OIE (2016). Fowl Pox. Manual of Diagnostc Tests and Vaccines for Terrestrial Animals.

[56] Tripathy D., Reed W., Calnek B.W., Barnes H., Beard C., McDougald L. (1997). Pox. Diseases of Poultry.

[57] Forrester D.J., Spalding M.G. (2003). Parasites and Diseases of Wild Birds in Florida.

[58] Williams R.A., Duch C.E., Pérez-Tris J., Benitez L. (2014). Polymerase chain reaction detection of avipox and avian papillomavirus in naturally infected wild birds: Comparisons of blood, swab and tissue samples. Avian Pathol..

[59] Malik Y.S., Raj Kumar S., Mahendra P.Y. (2019). Recent Advances in Animal Virology.

[60] Literak I., Kulich P., Robesova B., Adamík P., Roubalova E. (2009). Avipoxvirus in great tits (Parus major). Eur. J. Wildl. Res..

[61] Cunningham C.H., Hofstad M.S., Calnek B.W., Helmboldi C.F., Keid W.M., Yoder H.W. (1978). Avian pox. Diseases of Poultry.

[62] Murphy F.A., Gibbs E.P.J., Horzinek M.C., Studdert M.J., Gibbs E.P.J., Horzineck M.C., Studdert M.J. (1999). Poxviridae. Veterinary Virology.

[63] Haydar M.R., Ara M.S., Soma S.S., Rahman M.M., Rahman M.B., Rahman M.T. (2017). Isolation and molecular detection of turkeypox virus from turkey for the first time in Bangladesh. Bangladesh J. Veter. Med..

[64] Tripathy D.N., Calnek B.W. (1984). Pox. Diseases in Poultry.

[65] Jarmin S., Manvell R., Gough R.E., Laidlaw S.M., Skinner M.A. (2006). Avipoxvirus phylogenetics: Identification of a PCR length polymorphism that discriminates between the two major clades. J. Gen. Virol..

[66] Siddique A.B., Hossain F., Zinnah M. (2011). Determination of host specificity of pigeon pox and fowl pox viruses isolated from a field outbreak. Bulg. J. Vet. Med..

[67] Masola S.N., Mzula A., Kasanga C.J., Wambura P.N. (2014). Integration of reticuloendotheliosis virus in most of tanzanian fowl pox virus isolates is not attributed to imported commercial fowl pox vaccines. Br. Biotechnol. J..

[68] Robinson F.R., Twiehaus M.J. (1974). Historical note: Isolation of the avian reticuloendothelial virus (Strain T). Avian Dis..

[69] Hanson J., Howell J. (1979). Suspected reticuloendotheliosis in turkeys with cutaneous lesions reminiscent of fowl pox. Can. Veter. J. La Rev. Veter. Can..

[70] Wang J., Meers J., Spradbrow P.B., Robinson W.F. (2006). Evaluation of immune effects of fowlpox vaccine strains and field isolates. Veter. Microbiol..

[71] Witter R., Fadly A. (2003). Diseases of poultry, chapter 15: Subchapter—Reticuloendotheliosis. Diseases of Poultry.

[72] Payne L.N. (1998). Retrovirus-induced disease in poultry. Poult. Sci..

