# Supplementary material for: Molecular Detection of Reticuloendotheliosis Virus 5′ Long Terminal Repeat Integration in the Genome of Avipoxvirus Field Strains from Different Avian Species in Egypt
Source: Biology (Basel). 2020 Aug 31;9(9):257. doi: 10.3390/biology9090257 (PMC7563266; doi:10.3390/biology9090257)
Supplement: Supplementary file 1 [file biology-09-00257-s001.pdf]

Supporting information

Molecular Detection of Reticuloendotheliosis virus 5' Long Terminal Repeat Integration in the Genome of Avipoxvirus Field Strains from Different Avian Species in Egypt

Samah M. Mosad [1], Mohamed El-Tholoth 1,[2], Ali A. El-Kenawy [1], Lina Jamil M. Abdel-Hafez [3], Fatma A. El-Gohary [4], Hanem El-Sharkawy [5], Mona Mohieldin Elsayed [4], Ayman A. Saleh [6] and Ehab Kotb Elmahallawy [7,8*]

1 Department of Virology, Faculty of Veterinary Medicine, Mansoura University, 35516-Mansoura, Egypt. 1; dr.sama786@yahoo.com; (S.M.M); tholothvirol@mans.edu.eg (M.E); elkenawya@mansu.edu.eg (A.A.E)

2 Veterinary Science Division, Al Ain Men's Campus, Higher Colleges of Technology, 17155-Al Ain, Abu Dhabi, United Arab Emirates. 2; meltholoth@hct.ac.ae

3 Department of Microbiology and Immunology, Faculty of Pharmacy, October 6 University, October 6 City 12566, Giza, Egypt; Lina.jamil@ymail.com

4 Department of Hygiene and Zoonoses, Faculty of Veterinary Medicine, Mansoura University, Mansoura 35516, Egypt; dr.fatmagohary@gmail.com(F.A.E); dr.monamohy@yahoo.com (M.M)

5 Department of Poultry and Rabbit Diseases, Faculty of Veterinary Medicine, Kafrelsheikh University, Kafrelsheikh 33511, Egypt; hanem_amin@yahoo.com

6 Department of Animal Wealth Development, Genetics and Genetic Engineering, Faculty of Veterinary Medicine, Zagazig University, Zagazig 44519, Egypt; lateefsaleh@yahoo.com

7 Department of Biomedical Sciences, University of Leon, León 24071, Spain;

8 Department of Zoonotic diseases, Faculty of Veterinary Medicine, Sohag University, Sohag 82524, Egypt;

* Correspondence: Dr Ehab Kotb Elmahallawy, PhD, eehaa@unileon.es

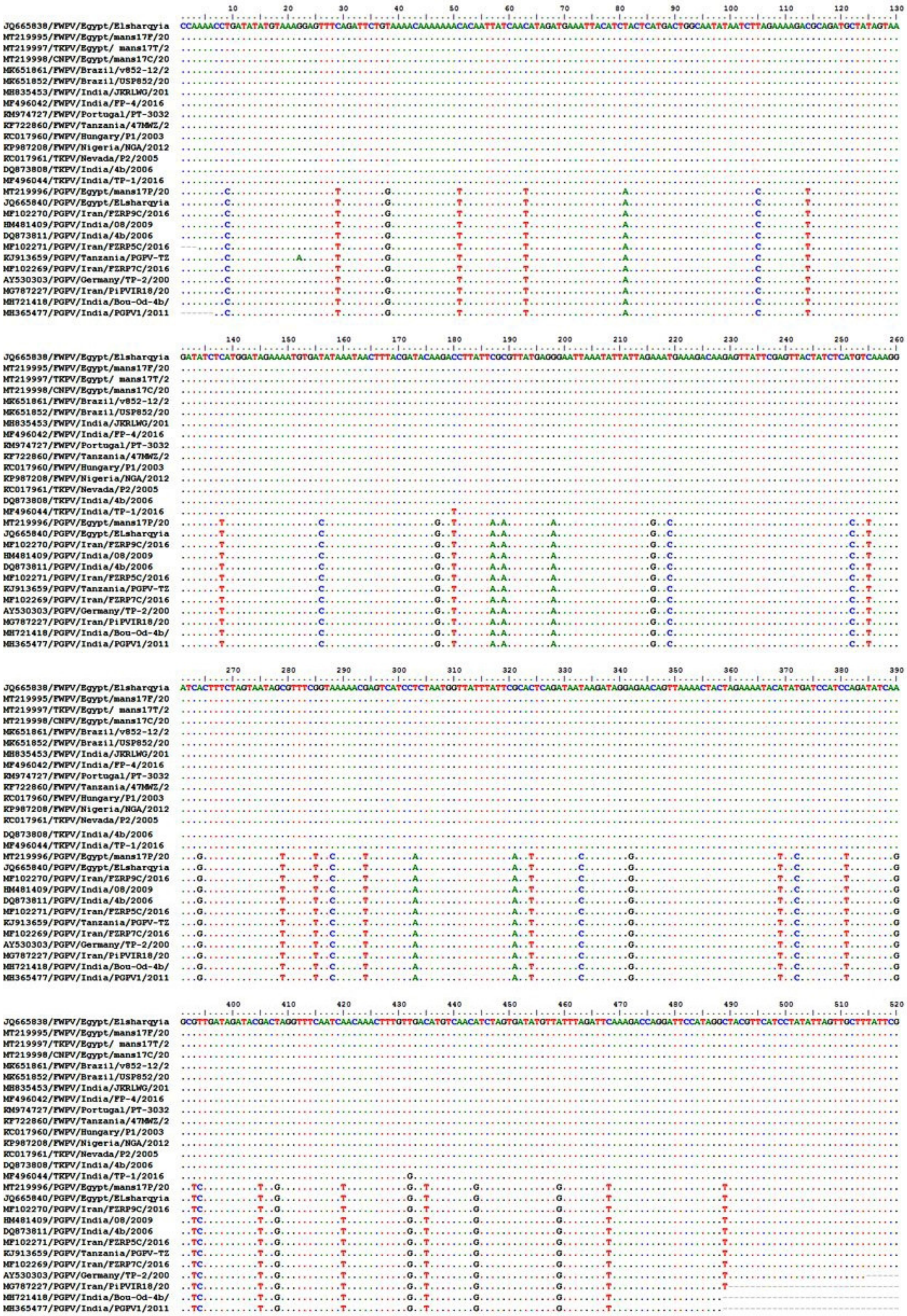

Supplementary Figure S1. The BioEdit multiple alignments of amplified *P4b* gene nucleotide sequences of our APVs isolates compared to other reference APVs sequences retrieved from GenBank. PGPV strains have 44 nucleotide substitutions in comparison with FWPV and TKPV strains.

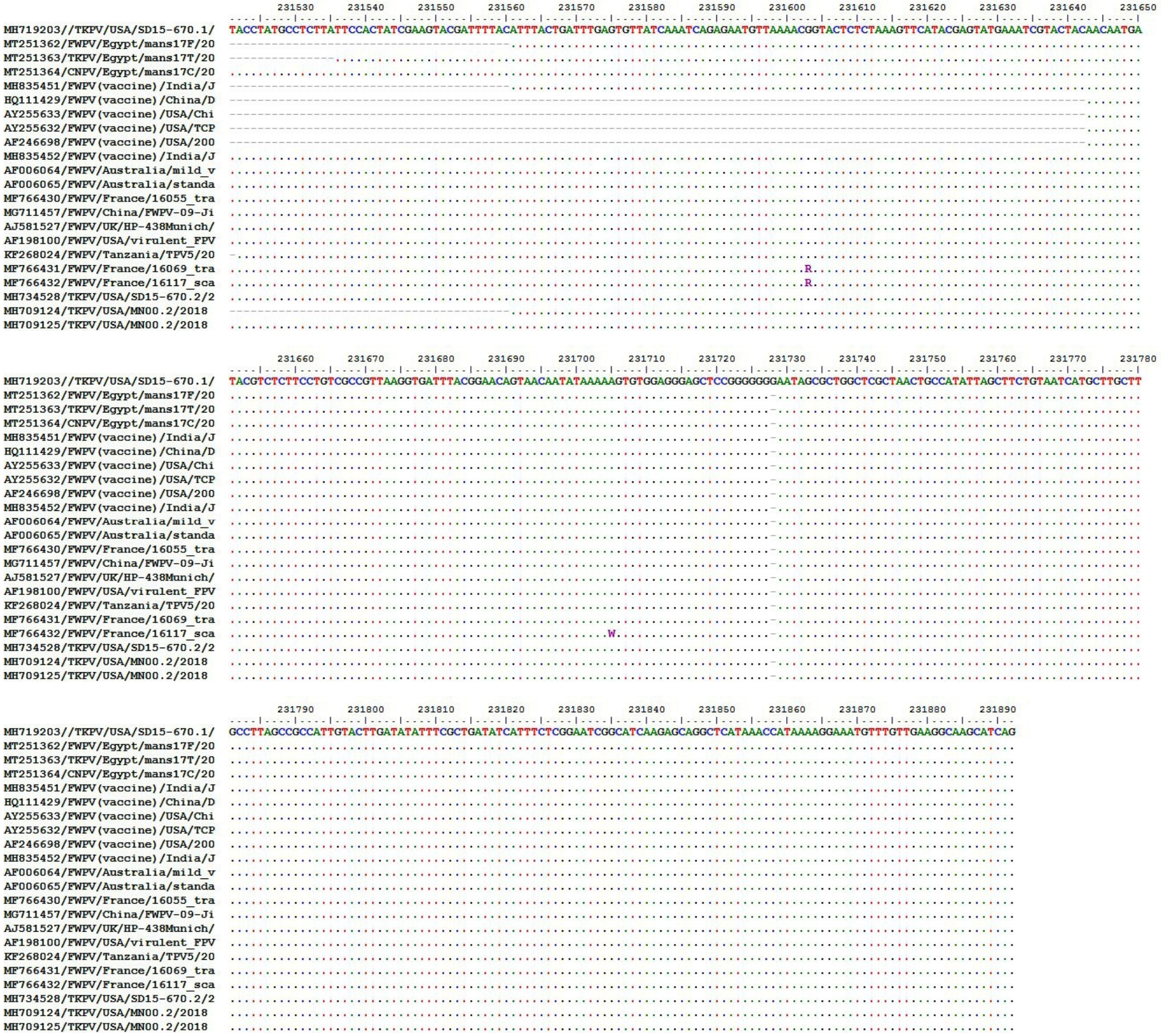

Supplementary Figure S2. The BioEdit multiple alignments of amplified REV-5′LTR nucleotide sequences detected in our APVs isolates in comparison to other reference REV-5’LTR sequences available in GenBank. All aligned sequences are identical to each other without any nucleotide substitution.
